# Honey, Propolis, and Royal Jelly: A Comprehensive Review of Their Biological Actions and Health Benefits

**DOI:** 10.1155/2017/1259510

**Published:** 2017-07-26

**Authors:** Visweswara Rao Pasupuleti, Lakhsmi Sammugam, Nagesvari Ramesh, Siew Hua Gan

**Affiliations:** ^1^Institute of Food Security and Sustainable Agriculture, Universiti Malaysia Kelantan, Campus Jeli, 17600 Jeli, Malaysia; ^2^Faculty of Agro-Based Industry, Universiti Malaysia Kelantan, Campus Jeli, 17600 Jeli, Malaysia; ^3^Human Genome Center, School of Medical Sciences, Universiti Sains Malaysia, Kubang Kerian, 16150 Kelantan, Malaysia

## Abstract

**Background:**

There are several health benefits that honeybee products such as honey, propolis, and royal jelly claim toward various types of diseases in addition to being food.

**Scope and Approach:**

In this paper, the effects of honey, propolis, and royal jelly on different metabolic diseases, cancers, and other diseases have been reviewed. The modes of actions of these products have also been illustrated for purposes of better understanding.

**Key Findings and Conclusions:**

An overview of honey, propolis, and royal jelly and their biological potentials was highlighted. The potential health benefits of honey, such as microbial inhibition, wound healing, and its effects on other diseases, are described. Propolis has been reported to have various health benefits related to gastrointestinal disorders, allergies, and gynecological, oral, and dermatological problems. Royal jelly is well known for its protective effects on reproductive health, neurodegenerative disorders, wound healing, and aging. Nevertheless, the exact mechanisms of action of honey, propolis, and royal jelly on the abovementioned diseases and activities have not been not fully elucidated, and further research is warranted to explain their exact contributions.

## 1. Introduction

Apiculture is the science and art of prolonging, sustaining, and retaining health by using products obtained from honeybee hives, such as honey, bee bread, bee venom, bee pollen, propolis, and royal jelly. Recent years have seen the fast application of bee products in both traditional and modern medicine. Currently, many studies are targeted toward investigating directed health benefits and pharmacological properties of bee products due to their efficacies, leading to the increasing development of nutraceuticals and functional food from these products. The concept of functional food refers to food that has the ability to promote better physiological or psychological health compared to traditional remediated and nutritional food. These effects positively contribute toward excellent health maintenance, well-being, and reduced chronic illness [1]. The present review focuses on the potential health benefits of bee products, including honey, propolis, and royal jelly.

Honey is a sweet liquid processed by the honey bee. Honey is recognized worldwide due to its high nutritive components that are beneficial for human well-being. It has been traditionally used by Egyptians, Greeks, Romans, and Chinese to heal wounds and diseases of the gut, including gastric ulcers. It has also been used as a remedy for cough, sore throat, and earaches [2]. In India, Lotus honey has been traditionally used to treat eye infections and other diseases. In addition to being used externally, honey is also used internally [3] as a functional food to provide energy and nourishment to enhance vital organs in the body [4]. This has been in practice since ancient times. The active components of honey, such as glucose, fructose, flavonoid, polyphenols, and organic acids, play an important role in its quality [5]. Honey is being produced in many countries all over the world and is recognized as an important medicine as well as energy-providing food due to its functional properties and nutritional values. Additionally, honey is well known for its biological, physiological, and pharmacological activities.

Propolis is generally known as the “bee glue”, which is a generic name that refers to the resinous substance accumulated by the bees from different types of plants. The word “propolis” is derived from Greek to mean defense for “pro” and city or community for “polis”, or the beehive, in other words [6]. Propolis functions in sealing holes and cracks and for the reconstruction of the beehive. It is also used for smoothing the inner surface of the beehive, retaining the hive's internal temperature (35°C), preventing weathering and invasion by predators. Furthermore, propolis hardens the cell wall and contributes to an aseptic internal environment. Propolis generally becomes soft and sticky upon heating [7]. It also possesses a pleasant smell. Propolis and its extracts have numerous applications in treating various diseases due to its antiseptic, anti-inflammatory, antioxidant, antibacterial, antimycotic, antifungal, antiulcer, anticancer, and immunomodulatory properties.

Royal jelly, a white and viscous jelly-like substance, is a form of hypopharyngeal and mandibular gland secretion from the worker bees. It is also known as a “superfood” that is solely consumed by the queen bee. Royal jelly is also fed to the honeybee larvae upon hatching and helps to nurture the brood [8]. It is the exclusive nutriment offered to the immature young larvae in their first 2-3 days of maturation besides being used as a food specifically for the queen bee throughout her entire life cycle. Royalactin is the main compound in royal jelly that allows the morphological change of a larva into the queen bee [9]. This superfood is the main reason for the longevity of the queen bee compared to the other bees. Royal jelly is widely used as a dietary nutritional complex to help combat various chronic health conditions. Furthermore, it is one of the profitable remedies for human beings in both traditional and modern medicine. Many pharmacological activities such as antibacterial, antitumor, antiallergy, anti-inflammatory, and immunomodulatory effects have also been attributed to it.

## 2. Chemical Composition of Honey, Propolis, and Royal Jelly

Honey is also known as a supersaturated sugar solution. Natural honey is composed of 82.4% carbohydrates, 38.5% fructose, 31% glucose, 12.9% other sugars, 17.1% water, 0.5% protein, organic acids, multiminerals, amino acids, vitamins, phenols, and a myriad of other minor compounds. In addition, honey consists of minor amounts of bioactive components, including phenolic acid, flavonoid, and *α*-tocopherol [10]. Honey constituents with health benefits include phenolic acids, flavonoids, ascorbic acid, proteins, carotenoids, and certain enzymes, such as glucose oxidase and catalase [11].

Propolis is the third most important component of bee products. It is composed mainly of resin (50%), wax (30%), essential oils (10%), pollen (5%), and other organic compounds (5%) [12]. Phenolic compounds, esters, flavonoids, terpenes, beta-steroids, aromatic aldehydes, and alcohols are the important organic compounds present in propolis [13]. Twelve different flavonoids, namely, pinocembrin, acacetin, chrysin, rutin, luteolin, kaempferol, apigenin, myricetin, catechin, naringenin, galangin, and quercetin; two phenolic acids, caffeic acid and cinnamic acid; and one stilbene derivative called resveratrol have been detected in propolis extracts by capillary zone electrophoresis [14]. Propolis also contains important vitamins, such as vitamins B1, B2, B6, C, and E and useful minerals such as magnesium (Mg), calcium (Ca), potassium (K), sodium (Na), copper (Cu), zinc (Zn), manganese (Mn), and iron (Fe). A few enzymes, such as succinic dehydrogenase, glucose-6-phosphatase, adenosine triphosphatase, and acid phosphatase, are also present in propolis [15].

Royal jelly consists of water (50%–60%), proteins (18%), carbohydrates (15%), lipids (3%–6%), mineral salts (1.5%), and vitamins [16]. Based on modern spectrometric analysis, approximately 185 organic compounds have been detected in royal jelly. Royalactin is the most important protein present in royal jelly. In addition, royal jelly is composed of a significant number of bioactive compounds, including 10-hydroxy-2-decenoic acid (HAD), which has some immunomodulatory properties [17]. Fatty acid, proteins, adenosine monophosphate (AMP) N1 oxide, adenosine, acetylcholine, polyphenols, and hormones such as testosterone, progesterone, prolactin, and estradiol are other useful bioactive components reported to be present in royal jelly [18].

## 3. Bioactive Compounds in Honey, Propolis, and Royal Jelly

Honey, propolis, and royal jelly are highly rich in bioactive compounds (Table 1). Essential and nonessential compounds, such as polyphenols and vitamins occurring naturally as part of food chains, are considered bioactive. These compounds are naturally present in food and confer useful health benefits. Phenolic compounds are bioactive compounds. Phenols are defined as organic compounds with an aromatic ring that is chemically bonded to one or additional hydrogenated substituents in the presence of corresponding functional derivatives [19].

**Figure 1:**
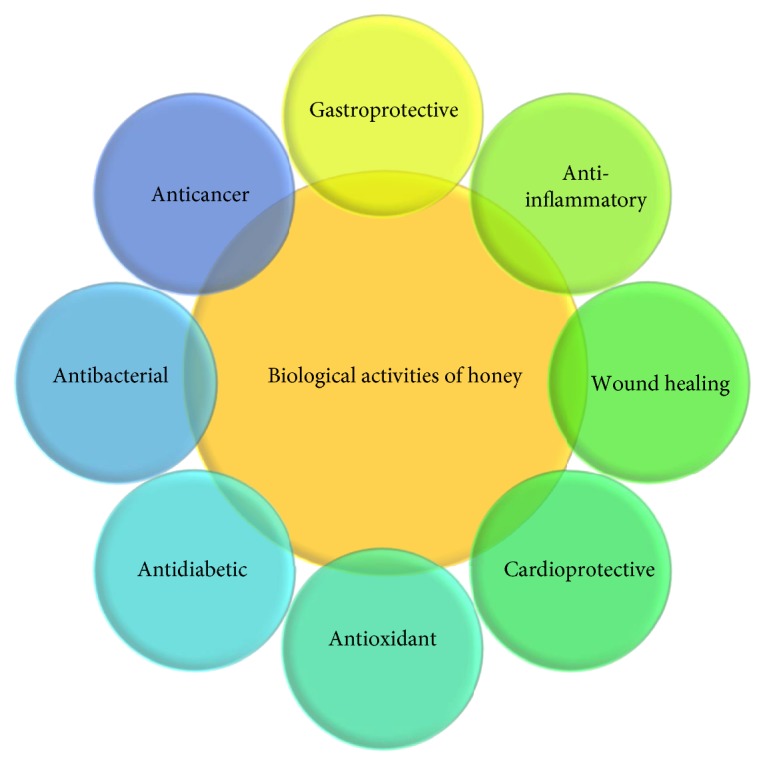
Various types of biological activities of honey products.

In honey, propolis, and royal jelly, phenolic compounds are commonly present as flavonoids [20]. Various phenolic compounds contribute to the functional properties of bee products, including their antioxidant, antimicrobial, antiviral, anti-inflammatory, antifungal, wound healing, and cardioprotective activities [21].  Figure 1 summarizes the important biological efficacies of bee products.

## 4. Health Benefits of Honey

### 4.1. Wound Management

Honey has traditionally been used to treat wounds, insect bites, burns, skin disorders, sores, and boils. Scientific documentation of the wound-healing capabilities of honey validates its efficacy as a promoter of wound repair and an antimicrobial agent [37]. Honey promotes the activation of dormant plasminogen in the wound matrix, which results in the dynamic expression of the proteolytic enzyme. Plasmin causes blood clot retraction and fibrin destructions. It is an enzyme that breaks down fibrin clots with attached dead tissues in the wound bed [38].

Clinical evidence supporting the effectiveness, specificity, and sensitivity of honey in wound care indicates that the performance of conventional and modern wound care dressing is inferior to that using honey [39]. Certain cases have shown that honey stimulates wound-healing properties even in infected wounds that do not respond to antiseptics or antibiotics and wounds that have been infected with antibiotic-resistant bacteria, such as methicillin-resistant *Staphylococcus aureus* (MRSA) (Natarajan et al. 2001). Honey also aids autolytic debridement and accelerates the growth of healthy granulated wound bed [40].

Malodor is a general attribute of severe wounds caused by anaerobic bacterial species belonging to *Bacteroides* spp. and *Peptostreptococcus* spp. [41]. Malodourous compounds, such as ammonia, amines, and sulfur, are produced by bacteria during the metabolism of amino acids from putrefied serum and tissue proteins. These compounds are replaced by lactic acids as honey dispenses a substantial amount of glucose, a substrate metabolized by bacteria in preference to amino acids [42]. The therapeutic effects observed after honey application include fast healing, wound cleansing, clearance of infection, tissue regeneration, minimized inflammation, and increased comfort during dressing due to lower extent of tissue adhesion [43].

### 4.2. Pediatric Care

Honey also controls skin damage near stomas, such as ileostomy and colostomy, by enhancing epithelialization of the afflicted skin surface [44]. Honey has a beneficial effect on pediatric dermatitis caused by excessive use of napkins and diapers, eczema, and psoriasis. The effect of honey mixed with beeswax and olive oil was investigated on patients with psoriasis or atopic dermatitis condition. A clinical trial showed that a mixture containing honey was extremely well tolerated and caused significant improvements. Honey consists of various nitric oxide metabolites, which reduce the incidence of skin infection in psoriasis [45].

### 4.3. Diabetic Foot Ulcer (DFU)

Consumption of honey is a low-cost and effective therapy for the treatment of DFU. DFU is often complicated by microbial infections and slows the healing process. Apart from the infection, symptoms such as pain, swelling, and redness might not be present for diabetic peripheral neuropathy patients due to their reduced immune response, which further complicates the diagnosis [46]. A review indicated that using honey for the treatment of venous ulcers yielded positive outcomes with good acceptance rates from the patients [47]. Honey is used in wound management and is effective among patients with locally infected wounds, DFU, Charcot foot ulcerations, and complex comorbid conditions that have failed hospital management [48]. In addition, there is excellent tolerability and minimal trauma to the wound bed in the presence of honey.

### 4.4. Gastrointestinal (GI) Disorder

Natural honey is composed of enzymes that facilitate the absorption of molecules, such as sugars and starch. The sugar molecules in honey are in a form that can be easily absorbed by the body. Honey also provides some nutrients, such as minerals, phytochemicals, and flavonoids, that aid digestive processes in the body [49]. Pure honey has bactericidal properties against pathogenic bacteria and enteropathogens, including *Salmonella* spp., *Escherichia coli*, *Shigella* spp., and many other Gram-negative species [50].

The gastrointestinal tract (GIT) contains many important beneficial microbes. For example, *Bifidobacteria* is one of the microorganisms present primarily for the sustenance of a healthy GI system. It has been suggested that consuming foods rich in probiotics can increase the population of *Bifidobacteria* in the GIT. The biological activities and development of this bacteria are further enhanced in the presence of prebiotics. Studies have shown that natural honey contains high amount of prebiotics [51]. Some in vitro and in vivo experimental trials on honey have reported it as a prominent dietary supplement that hastens the growth of *Lactobacillus* and *Bifidobacteria* and catalyzes their probiotic potency in the GIT [52, 53]. Under in vitro conditions, prebiotic ingredients in honey such as inulin, oligofructose, and oligosaccharides promoted the increase in the numbers of *Lactobacillus acidophilus* and *L. plantarum* by 10–100 folds, which was beneficial for the intestinal microbiota [54].

### 4.5. Oral Health

Honey is useful for the treatment of many oral diseases, including periodontal disease, stomatitis, and halitosis. In addition, it has also been applied for the prevention of dental plaque, gingivitis, mouth ulcers, and periodontitis. The antibacterial and anti-inflammatory properties of honey can stimulate the growth of granulation tissue, leading to the repair of damaged cells [55]. *Porphyromonas gingivalis* is a Gram-negative bacteria that causes periodontitis. Honey exerts antimicrobial activity against this anaerobic bacteria and prevents periodontal disease [56]. Inflammation of mucous membranes in the mouth (stomatitis) may induce redness and swelling of oral tissues and cause distinct and painful ulcers. Honey penetrates into the tissues very quickly and is effective against stomatitis [57, 58]. Halitosis is an oral health condition that causes malodorous breath. Most of the odor in the oral cavity is caused by the activity of degrading microbes [59]. A recent study has reported that honey consumption ameliorates halitosis due to its strong antibacterial activity resulting from its methylglyoxal component [60].

### 4.6. Pharyngitis and Coughs

Pharyngitis, commonly known as sore throat, is an acute infection induced by *Streptococcus* spp. in the oropharynx and nasopharynx [61]. In addition to streptococci, viruses, nonstreptococcal bacteria, fungi, and irritants such as chemical pollutants may also cause sore throat. Manuka honey is effective for treating sore throat with its anti-inflammatory, antiviral, and antifungal properties. Honey coats the inner lining of the throat and destroys the harmful microbes while simultaneously soothing the throat [62, 63].

A survey has demonstrated that honey is superior to other treatments for cough induced by upper respiratory infections, including dextromethorphan and diphenhydramine [64]. The antioxidant and antimicrobial properties of honey aided in minimizing persistent cough and ameliorated sleep for both children and adults following honey intake (2.5 ml). A comparative study on children with different natural products reported that honey was found to be the widely used remedy for pneumonia 82.4% [65].

### 4.7. Gastroesophageal Reflux Disease

Gastroesophageal reflux disease (GERD) is a mucosal infection caused by contents of abnormal gastric reflux into the esophagus and even the lungs. Symptoms of GERD include heartburn, inflammation, and acid regurgitation. Consumption of honey helps this condition by coating the esophagus and stomach lining, thus preventing the upward flow of food and gastric juice. Honey can further stimulate the tissues on the sphincter to assist in their regrowth and finally reduce the chances of acid reflux [66].

### 4.8. Dyspepsia, Gastritis, and Peptic Ulcer

Dyspepsia is a chronic disease in which the GI organs, mainly the stomach and first part of the small intestine, function abnormally. It is a disease that causes epigastric pain, heartburn, bloating, and nausea as symptoms. Dyspepsia is the preliminary symptom of peptic ulcer which could eventually cause cancer. Gastritis refers to the irritation and inflammation of the lining of the stomach wall. Peptic ulcer denotes erosions or open painful ulcers on the lining of the stomach or duodenum. Honey have been identified as a potent inhibitor for gastritis and the peptic ulcer causing agent, *Helicobacter pylori* (*H. pylori*) [67]. Clinical surveys have shown that honey decreased the secretion of gastric acid and increased the healing effect. Thus, honey is taken as a dietary supplement for its antibacterial properties and protective effect [68]. The high sugar content and low pH in honey are the results of glucose oxidative conversion to gluconic acid by glucose oxidase. This mechanism releases hydrogen peroxide, which functions as an antibacterial agent. Glucose oxidase also acts on fibroblasts and epithelial cell activators required for the healing of ulcers caused by *H. pylori* [51].

### 4.9. Gastroenteritis

Gastroenteritis, known as stomach or gastric flu, causes inflammation of the digestive tract. This condition may be due to foodborne, waterborne, and person-to-person spread of infectious agents. The symptoms of gastroenteritis include dehydration, watery diarrhea, bloating, abdominal cramps, and nausea. There are many infectious agents, such as *Salmonella*, *Shigella*, and *Clostridium*, that can cause this condition [69]. A clinical study by Abdulrahman, 2010, has reported the treatment of infantile gastroenteritis using honey. The study found that replacing the glucose in standard electrolyte oral rehydration solution (ORS) with honey reduced the recovery time of patients with gastroenteritis because the high sugar content in honey boosts electrolyte and water reabsorption in the gut [70].

### 4.10. Constipation and Diarrhea

Chronic constipation is a common and multifarious illness characterized by intolerable defecation (irregular stools and difficult stool passage). Difficult stool passage includes symptoms such as straining, hard to expel stool, a sense of incomplete evacuation, hard or lumpy stools, and prolonged time to pass stool [71]. Diarrhea is defined as a high frequency of bowel movements with watery stool. Honey has minimized the pathogenesis and duration of viral diarrhea compared to conventional antiviral therapy [72]. In another case, people diagnosed with inflammatory bowel syndrome (IBS) experiencing severe diarrhea or constipation, bloating, and stomach discomfort was successfully treated with raw Manuka honey on an empty stomach [73].

### 4.11. Liver and Pancreatic Diseases

Honey helps to soothe pain, balance liver systems, and neutralize toxins. Complications in the liver system can be attributed to oxidative damage. Honey exhibits antioxidant activities that have a potential protective effect on the damaged liver. A study on paracetamol-induced liver damage rats showed that the antioxidant and hepatoprotective activity of honey minimized liver damage [74]. Honey, which has a 1 : 1 ratio of fructose to glucose, may help to promote better blood sugar level, which is useful for those suffering from fatty liver disease since it provides adequate glycogen storage in liver cells. Insufficient glycogen storage in the liver releases stress hormones that impair glucose metabolism over time. Impaired glucose metabolism leads to insulin resistance and is the main factor of fatty liver disease. Another study reported significant reduction in blood glucose levels after treatment with Tualang honey [75, 76].

### 4.12. Metabolic and Cardiovascular Health

Natural wild honey exerts cardioprotective and therapeutic impacts against epinephrine-induced cardiac disorders and vasomotor dysfunctions. A harmonized relationship between radical scavenging activity and the total phenolic content of honey has been observed [77]. Honey intake showed a significant reduction in risk factors of metabolic and cardiovascular diseases. Honey exhibits cardioprotective effects such as vasodilation, balancing vascular homeostasis, and improvements in lipid profile [78]. Flavonoids in honey improves coronary vasodilation, decreases the ability of platelets to form clots, prevents oxidation of low-density lipoproteins (LDL), increases high-density lipoproteins (HDL), and improves endothelial functions [79].

A study conducted to compare the metabolic response of honey has indicated its ameliorative effects against metabolic syndromes (MetS) [80]. MetS is denoted by hyperglycemia, hypertension, abdominal obesity, dyslipidemia, and intensified adaptability towards diabetes, kidney, and heart diseases. Polyphenols in honey reduce atherosclerotic lesions through the downregulation of inflammatory and angiogenic mechanisms [81]. A clinical study conducted on patients with hyperlipidemia showed that honey decreased total cholesterol (TC) and noticeably prevented the rise in plasma glucose levels. Nitric oxide (NO) is a metabolite present in honey that also has cardioprotective functions [82].

### 4.13. Cancer and Oncogenesis

#### 4.13.1. Breast Cancer

Imbalance in estrogen signaling pathways and propagating levels of estrogens have important roles in breast cancer growth and propagation [83]. Treatments for breast cancer are associated with targeting the estrogen receptor (ER) signaling pathway. Phytoestrogens are a subclass of phytochemicals with a common structure to the mammalian estrogen that enables them to bind to estrogen receptors. Several experimental studies have investigated the efficiency of honey in modulating the ER signaling pathway [84]. Another study has shown that honey has biphasic activity in MCF-7 cells. This biphasic activity of honey is represented by an antiestrogenic effect at lower concentrations and an estrogenic effect at higher concentrations, which is caused when phytoestrogens bind to estrogen receptors [85]. Moreover, quercetin has been reported to induce apoptotic effects through ER *α*- and ER *β*-dependent mechanisms. On the other hand, cytotoxic activities of Tualang honey in human breast cancer cells were demonstrated by elevated secretion of lactate dehydrogenase (LDH) and further illustrated the cytotoxic properties of honey. The study also showed that honey only exerts cytotoxic effects on breast cancer line and not on nonmalignant breast cells. Therefore, this indicates that Tualang honey shows highly specific and selective cytotoxic effects towards breast cancer cell lines and has a good potential as a chemotherapeutic agent [86].

#### 4.13.2. Liver Cancer

The most common type of liver cancer is hepatocellular carcinoma (HCC). The antitumor effects of honey on liver cancer cells have been investigated in various experimental studies. Treatment of HepG2 cells with honey minimized the amount of nitric oxide (NO) levels in the cells and decreased the HepG2 cell number greatly. This increased the overall antioxidant profile of the cells. The survival of HepG2 cells is promoted by reactive oxygen species (ROS), and adequate levels of ROS trigger cell proliferation and differentiation. Decreasing the amount of NO resulting from honey treatment supported this study. Thus, reduced ROS and enhanced antioxidant efficacy inhibit cancerous cell proliferation and lowered the number of HepG2 cells [84]. Another study done by Abdel Aziz et al. investigated the effects of honey on HepG2 cell lines. The report showed that honey exerted cytotoxic, antimetastatic, and antiangiogenic effects on HepG2 cells based on different concentrations [87].

#### 4.13.3. Colorectal Cancer

Most colorectal cancers begin as a polyp, which generally starts on the inner lining of the colon or rectum and grows towards the center. Some polyps are not dangerous but some will eventually grow into adenomas and can eventually result in cancer. A study [88] that investigated the chemopreventive effects of Gelam and Nenas monofloral honeys against colon cancer cell lines found that the honey inhibited proliferation of colon cancer cells. Hydrogen peroxide-induced inflammation in the colon cancer cells was used to examine the effect of honey. The results showed that honey curbed inflammation in the cancerous cells [88]. Another study was done to investigate the apoptotic effects of crude honey on colon cancer cell lines. The study confirmed the antiproliferative effect of honey in these cells. In addition, at high phenolic concentrations (such as those of quercetin and flavonoids), significant antiproliferative action against colon cancer cells was observed [89].

**Figure 2:**
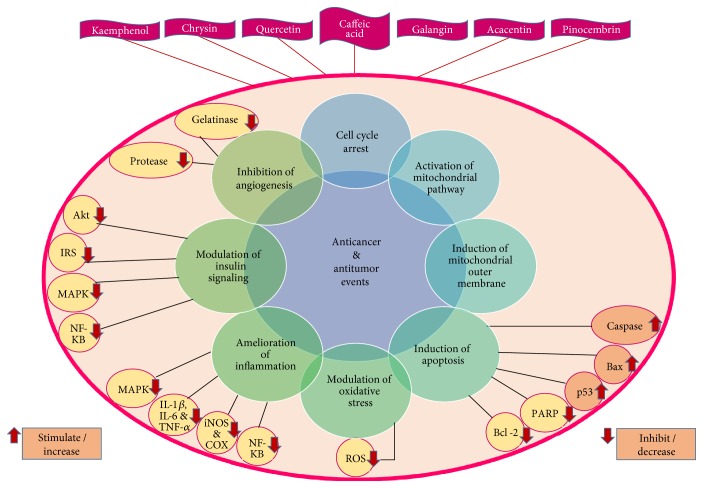
Molecular mechanisms responsible for anticancer and antitumor activities of honey products. IRS—insulin receptor substrate, MAPK—mitogen-activated protein kinase, NF-*κ*B—nuclear factor kappa B, IL-1*β*—interleukin-1 beta, IL-6—interleukin-6, TNF-*α*—tumor necrosis factor alpha, iNOS—inducible nitric oxide synthase, COX—cyclooxygenase, ROS—reactive oxygen species, Bcl-2—B-cell lymphoma-2, and PARP—poly (ADP-ribose) polymerases.

The molecular mechanisms resulting in the antiproliferative and anticancer effects of honey include cell cycle arrest, activation of mitochondrial pathway, induction of mitochondrial outer membrane permeabilization, induction of apoptosis, modulation of oxidative stress, reduction of inflammation, modulation of insulin signaling, and inhibition of angiogenesis in cancer cells (Figure 2). In addition, honey shows potential effects on cancer cell by modulating proteins, genes, and cytokines that promote cancer.

Several components of honey such as chrysin, quercetin, and kaempferol have been shown to arrest cell cycle at various phases such as G0/G1, G1, and G2/M in human melanoma, renal, cervical, hepatoma, colon, and esophageal adenocarcinoma cell lines. The mitochondrial pathway entails a chain of interactions between stimuli such as nutrients, physical stress, oxidative stress, and damage during major cancer treatments including chemotherapy and radiotherapy. These stimuli cause several proteins located within the intermembrane space (IMS) of the mitochondria, such as cytochrome c, to be released, which eventually culminates in the death of the cell. Flavonoids in honey are effective in activating the mitochondrial pathway and discharging proteins with potential cytotoxicity. Induction of mitochondrial outer membrane permeabilization (MOMP) is the most prevalent anticancer mechanism, which causes the leakage of proteins from the IMS and inevitably results in cell death. Honey induces MOMP in cancer cell lines by decreasing the mitochondrial membrane potential. Honey has also been documented for amplifying the apoptotic effect of tamoxifen by intensified depolarization of the mitochondrial membrane. Flavonoid constituents of honey, such as quercetin, have been shown to trigger MOMP and lead to cancer cell death [84].

Apoptosis is a programmed cell death functioning to control cell growth and remove damaged cells from the system. This process also involves MOMP and results in the discharge of IMS proapoptotic proteins such as cytochrome c to activate caspase cascades which results in further disruption of mitochondria and finally results in cancer cell death. Influence of honey on enzymes, genes, and transcription factors corresponding to apoptosis has been investigated. Poly (ADP-ribose) polymerases (PARP) are crucial enzymes involved in apoptosis and DNA repair. Inhibition of PARP activity renders the cells unable to repair damaged DNA and pass through the G2 and M phases of the cell cycle. Thus, cell cycle is arrested. Because DNA repair is impaired due to nonfunctioning PARP, the cells are being classified as damaged, and consequently, apoptosis activity may be augmented.

Inhibition of PARP activity by flavonoids in honey is a potential strategy for targeting cancers with defective DNA-damage repair. Bcl-2 and Bax are antiapoptotic and proapoptotic proteins, respectively. Bcl-2 is generally overexpressed in cancer. Tumor suppressor p53 is a transcription factor commonly inactivated in various types of tumors. It modulates transcription of genes involved in apoptosis [84, 90]. Honey enhances the upregulation of Bax and downregulation of Bcl-2. In addition, it activates caspases 3 and 9 and induces p53, thereby inhibiting cancer.

Low levels of ROS intensify cell proliferation while high levels lead to oxidative damage that contributes to various types of cancer. Regulation of redox homeostasis is vital for normal cell growth and proliferation. In this regard, honey is an influential antioxidant and free radical scavenger. The inhibitory effect of honey on cancer growth and proliferation is due to its ability to modulate oxidative stress. Honey exhibits anticancer properties via antioxidant or pro-oxidant mechanisms that are selectively dependent on the state of oxidative stress in the cancer cells. If cancer growth is rapid under high levels of ROS, honey acts as an antioxidant to prevent cancer cell growth by minimizing oxidative stress and scavenging the ROS. On the other hand, under low levels of ROS, it may also act as a pro-oxidant and promotes cancer cell growth by further generation of ROS and maximizing oxidative stress. Thus, the effects of honey on cancer cell death are different under different conditions [84].

Inflammation is a contributing factor for the dysregulation of physiological processes, which leads to various malignancies and cancers. Mitogen-activated protein kinase (MAPK) and nuclear factor kappa B (NF-*κ*B) are the two main pathways responsible for inflammatory response in cells. Activation of MAPK and NF-*κ*B activates proinflammatory genes and generates inflammatory proteins or cytokines. These include cyclooxygenase-2 (COX-2), C-reactive protein (CRP), lipoxygenase-2 (LOX-2), interleukins (IL-1*β*, IL-6), and TNF-*α*. These components play crucial roles in both angiogenesis and inflammatory responses corresponding to cancer. IL-1*β*, IL-6, and TNF-*α* are cytokines that trigger cancer cell proliferation by maintaining the inflammatory phenotype in the tumor microenvironment. On the other hand, cyclooxygenase-2 (COX-2) and inducible nitric oxide synthase (iNOS) yield essential endogenous factors responsible for the tumor progression. The actions of iNOS can be either inductive or inhibitory depending on the tumor types.

Biological responses which facilitate inflammation can promote tumorigenesis as severe inflammation is the major factor for the development of cancer cells. Treating and soothing of inflammation aid to suppress the configuration of malignant and benign tumors. Honey helps to reduce the promotion and tumorigenesis and progression of cancer by reducing the expression of MAPK and NF-*κ*B in cancerous cells. MAPK cascades are the main signaling pathways in the regulation of cell proliferation, survival, and differentiation. NF-*κ*B is a transcription factor which is vital in the regulation of immune responses, inflammation, and oncogenesis. NF-*κ*B translocation to the nucleus and reduced I*κ*B*α* degradation help to regulate the expression of genes involved in apoptosis and proliferation that are responsible for the development of cancer. Flavonoids found in honey have been shown to induce apoptosis and prevent the release of IL-1*β*, IL-6, TNF-*α*, iNOS, and COX-2 [84].

Tumors, malignancies, and cancers are usually enhanced by obesity and insulin-resistant type 2 diabetes mellitus. PI3K/Akt is an important pathway in insulin signaling. The PI3K/Akt pathway is also recognized in modulating substrates that are related to cellular growth, survival, and progression. Elevated levels of MAPK, NF-*κ*B, and insulin receptor substrate 1 (IRS-1) along with reduced levels of Akt expression have been actively linked to the development of insulin resistance. Honey components such as quercetin revive insulin resistance by increasing the expression of Akt while reducing the expression of IRS, MAPK, and NF-*κ*B. Modulation of insulin signaling by honey leads to anticancer activities [84].

Honey has debridement effects by boosting epithelialization and stimulates the development of granulation tissue through its angiogenic effect on the vasculature. Honey selectively stimulates angiogenesis in noncancer tissues through the production of hydrogen peroxide while inhibiting angiogenesis in cancer tissues. Honey has antiangiogenic effects that prevents the wound-healing response, reduces the viability of cancer cells, and lowers the incidence of metastasis by inhibiting the activities of gelatinase and protease. Honey prevents the development of cancer by blocking the three main stages of cancer formation known as initiation, proliferation, and progression [84].

## 5. Health Benefits of Propolis

### 5.1. Gastrointestinal Disorder

**Figure 3:**
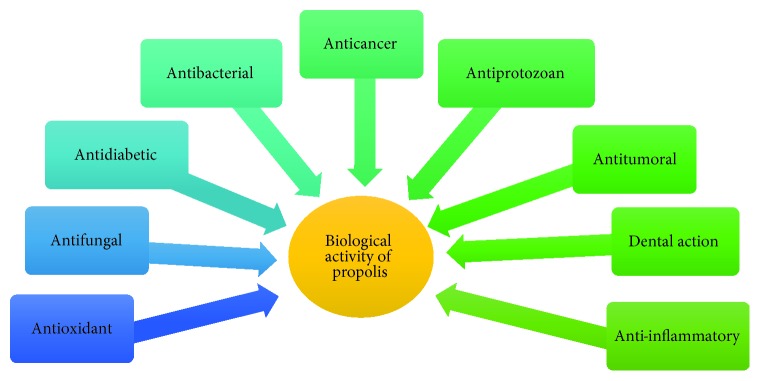
The biological activities of propolis.

Infection with parasites usually occurs upon contact with an infected surface. The symptoms of parasitic infection of the GI tract include abdominal pain, diarrhea, bloating, and nausea. Propolis has been reported to have several biological efficacies including anticancer, antioxidant, and anti-inflammatory activities (Figure 3). There are a few studies that reported the clinical use of propolis in the treatment of viral infections. In one study, the in vitro effect of propolis ethanolic extract on the growth and adherence of *Giardia duodenalis trophozoites* was evaluated [91]. Propolis was shown to inhibit growth and adherence of the trophozoites. It also promoted the detachment of these parasitic organisms. Its efficacy against giardiasis has also been reported in a clinical study whereby children and adults with giardiasis-given propolis showed a cure rate between 52% and 60%, whereas those given the conventional drug showed a 40% cure rate. Another experimental study showed that propolis has antihistaminergic, anti-inflammatory, antiacid, and anti-*H. pylori* activities that can be used to treat gastric ulceration [92].

### 5.2. Gynecological Care

Widespread causes of indicative vaginitis are bacterial vaginosis (BV) and vulvovaginal candidiasis (VVC). The depletion of *Lactobacillus* spp. in the vagina is a distinguished feature of vaginal infections. The infection is accompanied by an overgrowth of vaginal pathogens such as yeast-like fungi and an elevated vaginal pH. Diabetes patients are more prone to having vaginal infections caused by *Candida albicans*. A study conducted on the application of 5% aqueous propolis solution resulted in an improvement in vaginal well-being [93]. In addition to providing antibiotic and antimycotic actions, propolis provides early symptomatic relief due to its anesthetic properties. Thus, propolis may be used for Recurrent Vulvovaginal Candidiasis (RVVC) and can be an alternative option for patients who are unable to take antibiotics due to a concurrent pharmacological treatment. The effectiveness of propolis against conventional antifungal nystatin has shown satisfactory results. Propolis extract solution (PES) also show low toxicity in human cells and can be an alternative treatment for chronic vaginitis. In addition, PES has antifungal properties and it can be used as antibiofilm material for RVVC to counteract biofilm growth of *C. albicans* and resistance in antifungal drug [94].

### 5.3. Oral Health

The oral cavity has an abundant bacterial microflora and excessive bacterial growth may lead to several conditions such as oral diseases. Studies have shown that propolis may restrict bacterial-plaque development and periodontitis-causing pathogens because of its antibacterial properties [95]. Propolis solutions exert a selectively lower cytotoxic action on human gum fibroblasts compared to chlorhexidine. In addition to that, mouthwash containing propolis have shown effectiveness in healing surgical wounds. This encourages the use of propolis in solutions used as mouthwash [96]. Propolis solution can also be used to disinfect toothbrushes [97]. A 3% ethanolic extract of propolis toothpaste gel showed greater potency against gingivitis caused by dental plague in a group of patients [98]. Propolis extracts have also helped cure halitosis, a condition where an individual experiences unpleasant breath predominantly due to poor oral hygiene. Propolis toothpaste or mouthwash is used for their ability to reduce growth of bacterial plaque and pathogenic microflora that causes gingivitis and periodontitis. Thus, propolis also plays a role as a therapeutic agent [95].

### 5.4. Oncological Treatment

A study reported that propolis has potential towards human breast cancer treatment due to its antitumor activity by inducing apoptosis on human breast cancer cells. It also exhibits low or no toxicity towards normal cells due to its selectively toxic properties against tumor cells and is believed that propolis may become a prominent agent to treat breast cancer [99]. Another study investigating the effect of ethanolic extract of Algerian propolis on melanoma tumor growth has shown that galangin, a common flavonoid in propolis remarkably induced apoptosis and inhibited melanoma cells in vitro [100]. Turkish propolis has also been shown to exert a selective cytotoxic action on human lung cancer cells by inducing endoplasmic reticulum stress, apoptosis, and caspase activity and by reducing the mitochondrial membrane potential. This indicates that propolis is able to minimize the cancer cell proliferation [101].

### 5.5. Dermatological Care

Propolis is widely used in dermatological products such as creams and ointments. Its use in skin care products is based on its antiallergy, anti-inflammation, antimicrobial properties, and promotive action on collagen synthesis. A recent study comparing the effect of propolis and the conventional drug silver sulfadiazine showed that propolis notably decreased free radical activity in healing the wound beds which supported the repair process. A clinical study on acne patients using ethanolic extract propolis showed its high efficacy in the treatment of acne vulgaris [102]. Propolis also shows positive collagen metabolism in the wound during the healing process by increasing the collagen content of tissues [103]. A study demonstrated the use of propolis as an alternative therapy for wound healing to promote wound closure, especially under conditions such as human diabetic foot ulcer (DFU) [104].

**Figure 4:**
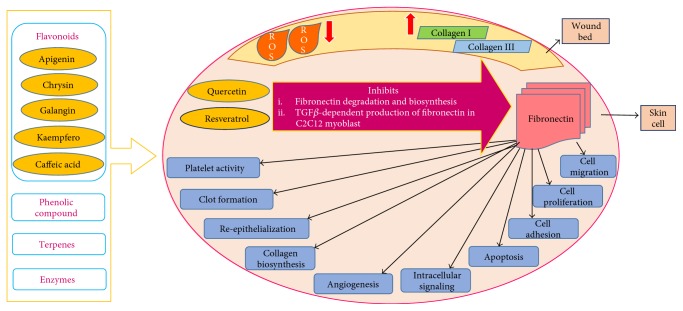
Molecular mechanism targeting wound-healing activity of propolis.

The molecular mechanisms responsible for the wound-healing activity of propolis is shown in Figure 4. Fibronectin (FN) is a multifunctional glycoprotein of high molecular weight, which influences the structural stability and functional properties of various organs and tissues (Stoffels, 2013). The fibronectin matrix and its accumulation are essential for cell migration, cell proliferation, cell differentiation, cell adhesion, apoptosis, cellular signaling, angiogenesis, collagen biosynthesis, re-epithelialization, clot formation, and platelet activity. Fibronectins are also important in the repair mechanisms for conditions such as glycoprotein intensified degradation, which leads to a defective cellular microenvironment and affliction in the structure of granulation tissues. This condition may prevent the wound from healing or inhibit the repair process. The accumulation of fibronectin in the extracellular space also modulates the secretion of other repairing components such as collagen type I and type III, tenascin, laminin, and fibrillin.

Propolis has demonstrated favorable effects in the wound-healing process such as antifungal and antibacterial activities due to its components such as flavonoids, phenolic compounds, terpenes, and enzymes. It also reduces the activity of free radicals (ROS) in the wound bed favoring the repair process. Propolis has also demonstrated great effects on collagen metabolism by increasing the amount of both type I and type III collagens in tissues. The reduction of ROS and accumulation of collagen aid in balancing the extracellular matrix and generating granulation tissues. Propolis is a potential apitherapeutic agent that is able to modify the metabolism of fibronectin by developing a fibrous network of extracellular matrix and inhibiting fibronectin disintegration. The active components in propolis such as quercetin and resveratrol inhibited fibronectin biosynthesis and TGF*𝛽*-dependent production of fibronectin, respectively, in C2C12 myoblasts. Both the components play important roles in regulating the expression of fibronectins. Studies have also shown that mobility and migration of epithelial cells are dependent on reduced fibronectin content in the extracellular matrix. Reduced amounts of this glycoprotein in propolis effectively treated wounds and produced granulation tissues. Therefore, the influence of propolis on fibronectin metabolism may alter the mechanism of wound healing [103]. Several health benefits of propolis related to gastrointestinal, gynecological care, oral health, skin care, and oncological treatments are tabulated in Table 2.

## 6. Health Benefits of Royal Jelly

**Figure 5:**
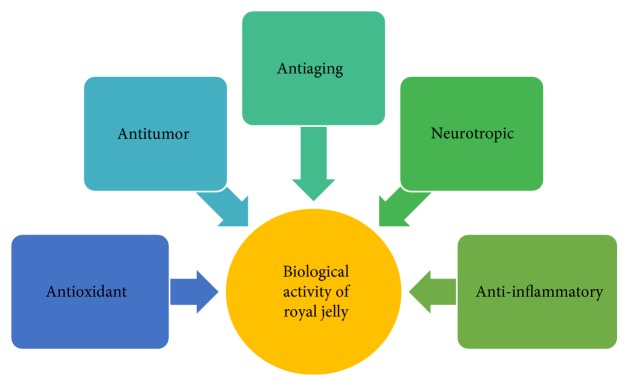
Different types of biological activities of royal Jelly.

Royal jelly is one of the honey bee products which have potential towards various human disease treatments.  Figure 5 depicts the biological activities of royal jelly as an antioxidant, antitumor, antiaging, neurotropic, and anti-inflammatory agent.

### 6.1. Reproductive Health

A randomized clinical study has reported that royal jelly is effective in reducing premenstrual syndrome [105]. A randomized clinical trial study reported the effectiveness of royal jelly in treatment of urinary problems and promotion of life quality in postmenopausal women [106]. Royal jelly has protective effects against Oxymetholone-induced reproductive toxin (OXM), which is an active steroid derived from testosterone as a defense mechanism. Recent studies have reported that royal jelly protects against the oxidative injuries in the mouse testes and that it contains spermatogenesis-stimulating compounds, which inhibit the production of proinflammatory cytokines [107]. Another study on male rabbits has indicated its positive effects on fertility, semen quality and output, and concentration of testosterone, total proteins, and glucose in the blood. The number of dead and abnormal sperm decreased with the reduction of biomarkers of oxidative stress [108]. Royal jelly has been traditionally used to treat menopause symptoms by rebalancing the hormonal concentration in the blood, decreasing follicle-stimulating hormones (FSH) and increasing the estrogen concentration in aged mice. A study showed that the changes in hormone levels resulting from royal jelly increased the amount of ovulated oocytes and their quality in aged rats [109].

**Figure 6:**
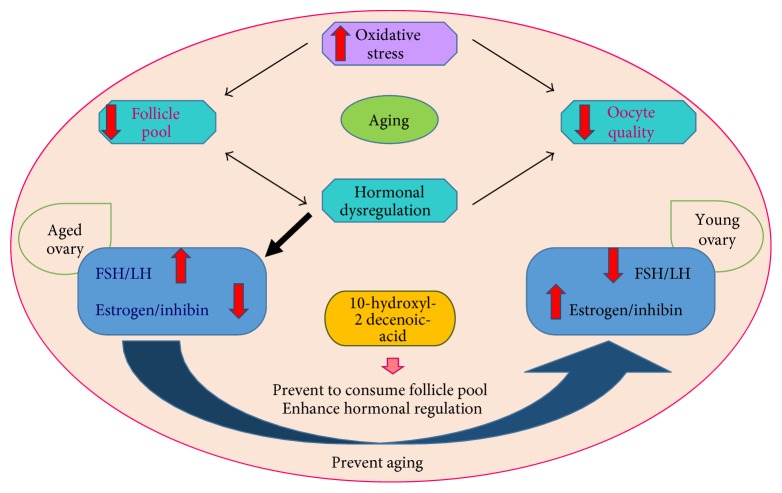
Molecular mechanism responsible for the antiaging activity of royal jelly.

The molecular mechanisms responsible for the antiaging activity of royal jelly are shown in Figure 6. The quality of oocytes decreases with age and the depleted follicle pool hastens hormonal dysregulation. This hormonal dysfunction is responsible for the reduction in ovarian follicle size and oocyte quality. Oxidative stress is the main cause of aging. Increased oxidative stress and continuous ovulation causes loss of antioxidants such as SOD, catalase, and glutathione S-transferase (GST). It also minimizes the size of the follicle pool and oocyte quality. Oxidative stress is controlled by glutathione (GSH), glutathione S-transferase (GST), Glutathione S-Transferase Theta 1 (GSTT1), Bax, and Bcl-2. GSH, GST, and GSTT1 are direct ROS scavengers, which play a vital role in removing oxidative stress from the cell. Higher expression of Bax and lower expression of Bcl-2 also promote aging and reduces oocyte quality.

FSH and luteinizing hormone (LH) are the hormones involved in the aging process. The amount of FSH and LH is controlled by estrogen (E2) and inhibin from the ovarian cells. Reduction of the follicle pool size results in an inadequate release of estrogen and inhibin, which results in a rise in FSH levels. This process then aids in the reduction of the follicle pool size and affects oocyte quality. This process promotes aging in the ovaries. In young ovarian cells, higher amount of estrogen (E2) and inhibins are needed to decrease the level of FSH and LH. This adaptation can be overcome by antiaging therapies such as supplemental consumption of royal jelly. The major active component present in royal jelly is 10-hydroxyl-2-decenoic acid. This compound enhances the synthesis of ovulation hormones, maintaining a lower expression of FSH and LH in young ovarian cells. It is also efficient in preventing the depletion of follicle pool and in enhancing hormonal regulation. Thus, royal jelly helps in preventing the aging process and is an influential antiaging product [109].

### 6.2. Neurodegenerative and Aging Diseases

Poor mental state and performance such as in the case of Alzheimer's disease (AD) are mostly experienced by elderly individuals due to aging. Royal jelly stimulates physical and mental functions for the elderly and increases their appetite and weight. A study showed that royal jelly exerted neuroprotective effects in AD [110]. The behavioral and neurochemical effect of royal jelly was chemically examined in aged rats. The study confirmed a better cognitive performance and increased the life span in the older animals that had been given royal jelly. Another study reported that royal jelly contains longevity-promoting factors and extends the lifespan in the nematode *Caenorhabditis elegans* [111]. Another study have also reported the improved mental health in human upon ingestion of royal jelly for six months [112]. A few studies on the health benefits of royal jelly are given in Table 3.

### 6.3. Wound Healing

Royal jelly enhances wound-healing activity. In both in vivo and in vitro wound-healing models, under the effect of royal jelly, human fibroblasts were able to migrate and increase levels of sphingolipids by decreasing the secretion and formation of collagen. Thus, royal jelly shortened the curing period of desquamated skin lesions [113]. Another study on the use of royal jelly have also exhibited protective action on human skin against ultraviolet B-induced photoaging by promoting collagen production [114]. Royal jelly dressing is also an effective way of treating diabetic foot ulcers besides standard treatments. This is due to its vasodilation effects around the affected wound, which can help to dilate the blood vessels to enhance blood flow. It also helps to prevents infections due to its antimicrobial activities [115].

## 7. Conclusion and Future Prospects

The present review focused on the potential health benefits of bee products such as honey, propolis, and royal jelly. These products are highly rich in active components such as flavonoids, phenolic acid, phenolic compounds, terpenes, and enzymes, which have biological functions in preventing some diseases and promoting good health. Honey, propolis, and royal jelly have distinct efficacies with significant nutritional properties and functional values. Thus, these bee products can be developed into potent apitherapeutic agents. However, some precautions need to be taken in case of allergens associated with bee products and in finding the right intake dosage. Hence, it is necessary to conduct further studies to determine the critical mechanisms related to the pharmacological activities of these bee products and the appropriate amounts that can be taken in order to obtain promising health benefits.

## Figures and Tables

**Table 1:** Important bioactive compounds in honey, propolis, and royal jelly.

Type of bee product	Bioactive compound	Chemical structure	Biological activity	References
Propolis	Phenolic compound: 2,2-dimethyl-8-prenylchromene	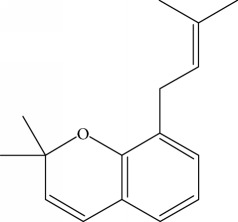	Antimicrobial	Viuda-Martos et al. [22]

Propolis	Phenolic compound: 4-hydroxy-3,5-diprenyl cinnamic acid (artepillin C)	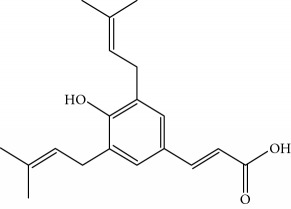	Antimicrobial, anti-inflammatory, anticancer	Viuda-Martos et al. [22]

Propolis	Phenolic compound: 3-prenyl cinnamic acid allyl ester	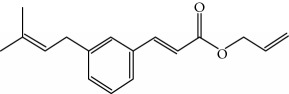	Antimicrobial	Viuda-Martos et al. [22]

Propolis	Phenolic compound: kaempferide	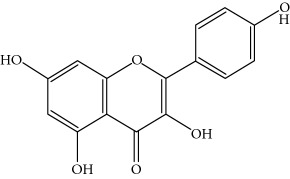	Antitumor, anticancer	Viuda-Martos et al. [22], [23]

Propolis	Phenolic compound: propolis benzofuran	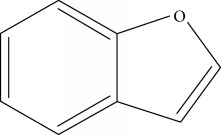	Antifungal	Viuda-Martos et al. [22], [23]

Propolis	Terpenoid: isocupressic acid, a labdane diterpenoid	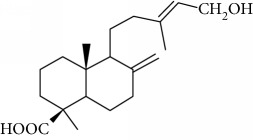	Antifungal	Viuda-Martos et al. [22] (Khalil & Sulaiman [23])

Propolis	Terpenoid:13C-symphyoreticulic acid, a clerodane diterpenoid	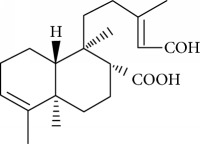	Antitumor	Viuda-Martos et al. [22], [24]

Propolis	Terpenoid: esters of long-chain fatty acids, (3-hydroxystearic acid (*n* = 11) procrim a; 3-hydroxystearic acid (*n* = 13), procrim b and a pentacyclic triterpenoid (lupeol))	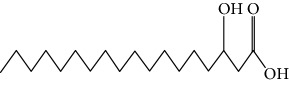	Antioxidant, antimicrobial, antitumor	(Salatino et al. [25]), Viuda-Martos et al. [22], (Huang et al. [13])

Propolis	Terpenoid: farnesol, a sesquiterpenoid	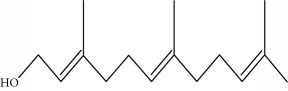	Antifungal	Viuda-Martos et al. [22], (Cotoras et al. [26])

Propolis, honey	Flavonoid: apigenin	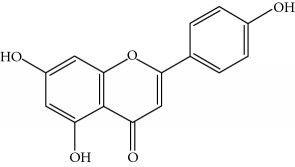	Antibacterial, anti-inflammatory	Viuda-Martos et al. [22], (Khalil & Sulaiman [23])

Honey, propolis	Flavonoid: acacetin	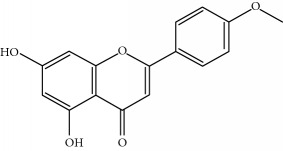	Antiallergy, anticancer	Viuda-Martos et al. [22], (Khalil & Sulaiman [23])

Honey, propolis	Flavonoid: quercetin	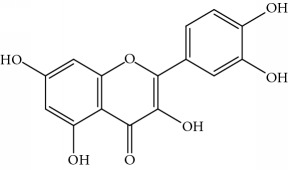	Anticancer, antiallergy, antibacterial, anti-inflammatory	Viuda-Martos et al. [22], (Khalil & Sulaiman [23])

Honey, propolis	Flavonoid: galangin	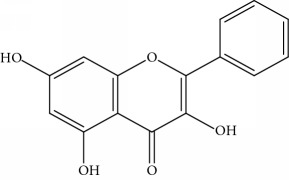	Anticancer, antioxidant	Viuda-Martos et al. [22], (Khalil & Sulaiman [23])

Honey, propolis	Flavonoid: pinocembrin	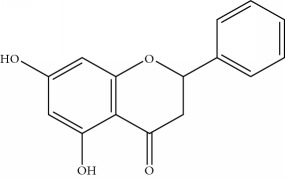	Antimicrobial, anticancer	Viuda-Martos et al. [22], (Khalil & Sulaiman [23])

Honey, propolis	Flavonoid: chrysin	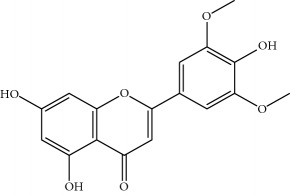	Antibacterial, anti-inflammatory, anticancer	Viuda-Martos et al. [22], (Khalil & Sulaiman [23])

Honey, propolis	Flavonoid: fisetin	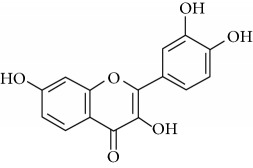	Antibacterial, antiallergy, anticancer	Viuda-Martos et al. [22], (Abubakar et al. [27])

Honey, propolis	Flavonoid: caffeic acid phenethyl ester	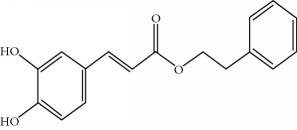	Antitumor, anticancer	Viuda-Martos et al. [22], (Khalil & Sulaiman [23])

Propolis, royal jelly	10-hydroxyl-2-decenoic acid	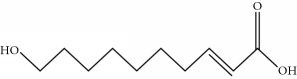	Antibiotic, antitumor	Izuta et al. [28]

Honey	Flavonoid: luteolin	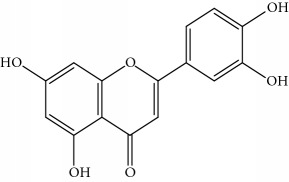	Antioxidant, anti-inflammatory, antitumor	Lin et al. [29], Mijanur et al. [30]

Honey	Flavonoid: pinobanksin	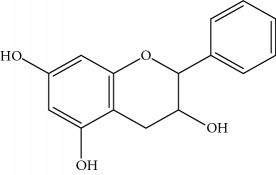	Antioxidant	Ajao et al. [31]

Honey	Flavonoid: hesperetin	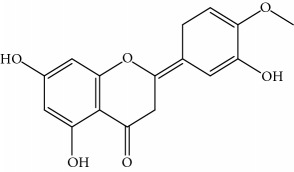	Antioxidant, anti-inflammatory	Kassim et al. [32], Mijanur et al. [30]

Honey	Flavonoid: naringenin	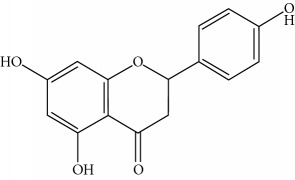	Neuroprotective, antioxidant	Badruzzaman Khan et al. [33], Mijanur et al. [30]

Honey	Flavonoid: genistein	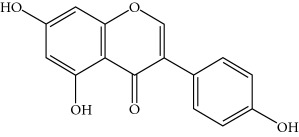	Anticancer	Tahir et al. [34], Mijanur et al. [30]

Honey	Phenolic acid: p-coumaric acid	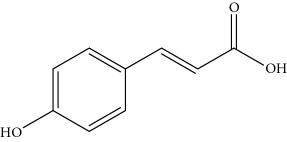	Antigenotoxic, neuroprotective	Vauzour et al. [35], Mijanur et al. [30])

Honey	Phenolic acid: gallic acid	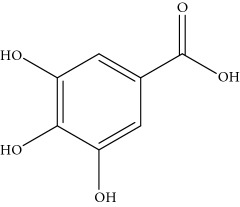	Antianxiolytic	Mansouri et al. [36], Mijanur et al. [30]

Honey	Phenolic acid: ellagic acid	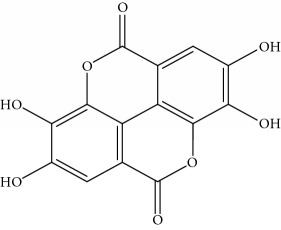	Antioxidant, chemopreventive, antiproliferative	Mijanur et al. [30]

Honey	Phenolic acid: ferulic acid	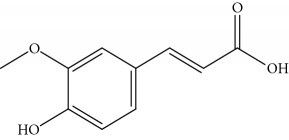	Antioxidant, anti-inflammatory, neuroprotective	Mijanur et al. [30]

Honey	Phenolic acid: syringic acid	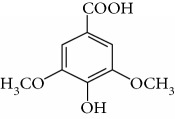	Antioxidant, anticancer	Mijanur et al. [30], Tahir et al. [34]

**Table 2:** Selected propolis activities according to the health benefits.

Health benefits	Propolis activity	Type of studies	Authors
GI disorder	Antiparasitic	Humans	Freitas et al. 2006 [91]
	Antiulceration	Animals	Paulino et al. 2015 [92]

Gynecological care	Antifungal	Human	Imhof et al. 2005 [93]
	Antifungal and antibiofilm	Human	Capoci et al. 2015 [94]

Oral health	Antibacterial	Laboratory	Pereira et al. 2011 [95]
	Daily mouthwash	Human	Jain et al. 2014 [96]
	Toothpaste disinfection	Laboratory	Bertolini et al. 2012 [97]
	Toothpaste against gingivitis	Human	Skaba et al. 2013 [98]
	Oral therapeutic agent	Human	Pereira et al. 2011 [95]

Oncology treatment	Anti-breast cancer	Human	Xuan et al. 2014 [99]
	Antimelanoma cancer	Animals	Benguedouar et al. 2015 [100]
	Anti-lung cancer	Human	Demir et al. 2016 [101]

Dermatology care	Acne vulgaris	Human	Ali et al. 2015 [102]
	Collagen metabolism	Animals	Olczyk et al. 2014 [103]
	Diabetic foot ulcer	Human	Henshaw et al. 2014 [104]

**Table 3:** Reports on health benefits of royal jelly.

Health benefits	Propolis activity	Type of studies	Authors
Reproductive care	Antioxidant	Animals	El-Hanoun 2012 [108]
	Hormone balance	Animals	Imai et al. 2012 [109]
	Antioxidative agent	Animals	Najafi et al. 2014 [107]
	Reduce premenstrual syndrome	Humans	Taavoni et al. 2014 [105]
	Postmenopausal treatment	Humans	Seyyedi et al. 2016 [106]

Neurodegenerative and aging disease	Longevity promoting	Animals	Honda et al. 2011 [111]
	Alzheimer's disease	Animals	Zamani et al. 2012 [110]
	Mental health	Human	Morita et al. 2012 [112]

Wound healing	Fibroblast migration	Animals	Kim et al. 2010 [113]
	Collagen production	Human	Park et al. 2011 [114]
	Vasodilatation	Human	Siavash et al. 2015 [115]
